# Supplementary material for: GET_PHYLOMARKERS, a Software Package to Select Optimal Orthologous Clusters for Phylogenomics and Inferring Pan-Genome Phylogenies, Used for a Critical Geno-Taxonomic Revision of the Genus Stenotrophomonas
Source: Front Microbiol. 2018 May 1;9:771. doi: 10.3389/fmicb.2018.00771 (PMC5938378; doi:10.3389/fmicb.2018.00771)
Supplement: Supplementary file 1 [file Presentation_1.pdf]

https://www.frontiersin.org/articles/10.3389/fmicb.2018.00771/full#supplementary-material

Supplementary material for:

Vinuesa P*, Ochoa-Sánchez LE and Contreras-Moreira B (2018).
GET_PHYLOMARKERS, a software package to select optimal orthologous clusters for phylogenomics and inferring pan-genome phylogenies, used for a critical geno-taxonomic revision of the genus *Stenotrophomonas*. *Front. Microbiol. 9:771. doi: 10.3389/fmicb.2018.00771*

https://www.frontiersin.org/articles/10.3389/fmicb.2018.00771/full

Published as part of the Research Topic on: **Microbial Taxonomy, Phylogeny and Biodiversity**: https://www.frontiersin.org/research-topics/5493/microbial-taxonomy-phylogeny-and-biodiversity

*Correspondence: Pablo Vinuesa, vinuesa[at]ccg.unam.mx

Pablo Vinuesa's ORCID: http://orcid.org/0000-0001-6119-2956
Bruno Contrera-Moreira's ORCID: https://orcid.org/0000-0002-5462-907X

Running title: **Estimating genome phylogenies with GET_PHYLOMARKERS**

The code described in this work is written in Bash, Perl and R for Linux/Unix/MacOS servers, and is freely available at https://github.com/vinuesa/get_phylomarkers under the GNU GPLv3 license. A docker image bundling it with GET_HOMOLOGUES is also available at https://hub.docker.com/r/csicunam/get_homologues/, which can be run on Windows machines.

Contents:

1. **Supplementary figures S1-S6**
2. **Supplementary tables S1 and S2**
3. **Supplementary code snippets, as examples on how to run a selection of the auxiliary scripts distributed in the GET_PHYLOMARKERS package**

1. Supplementary Figures

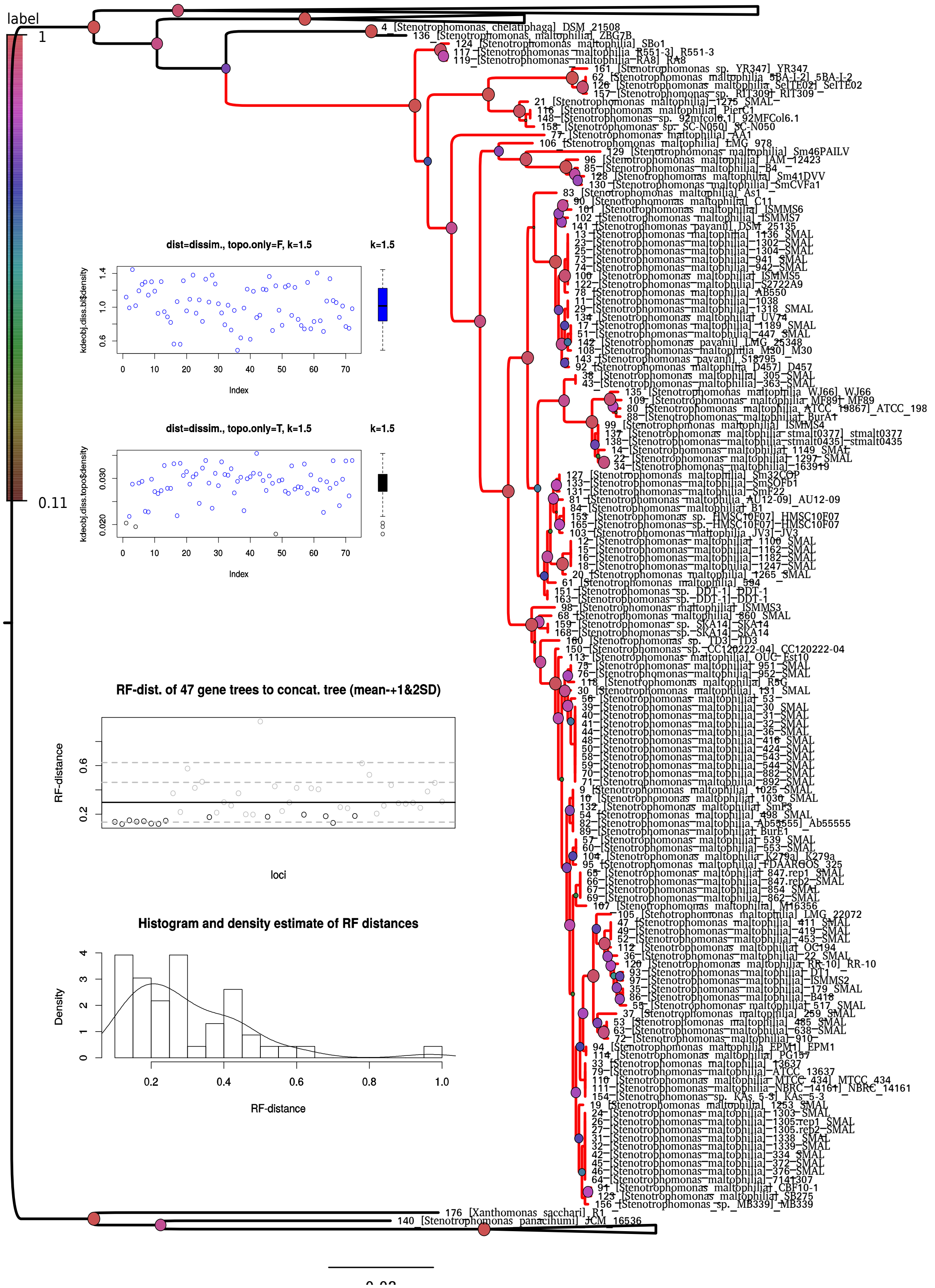

Figure S1. Maximum-likelihood phylogeny (FastTree) of the full set of reference genomes analyzed in this study (170 *Stenotrophomonas* spp. + 7 *Xanthomonas* spp.). Some clades collapsed for better readability of the tree.

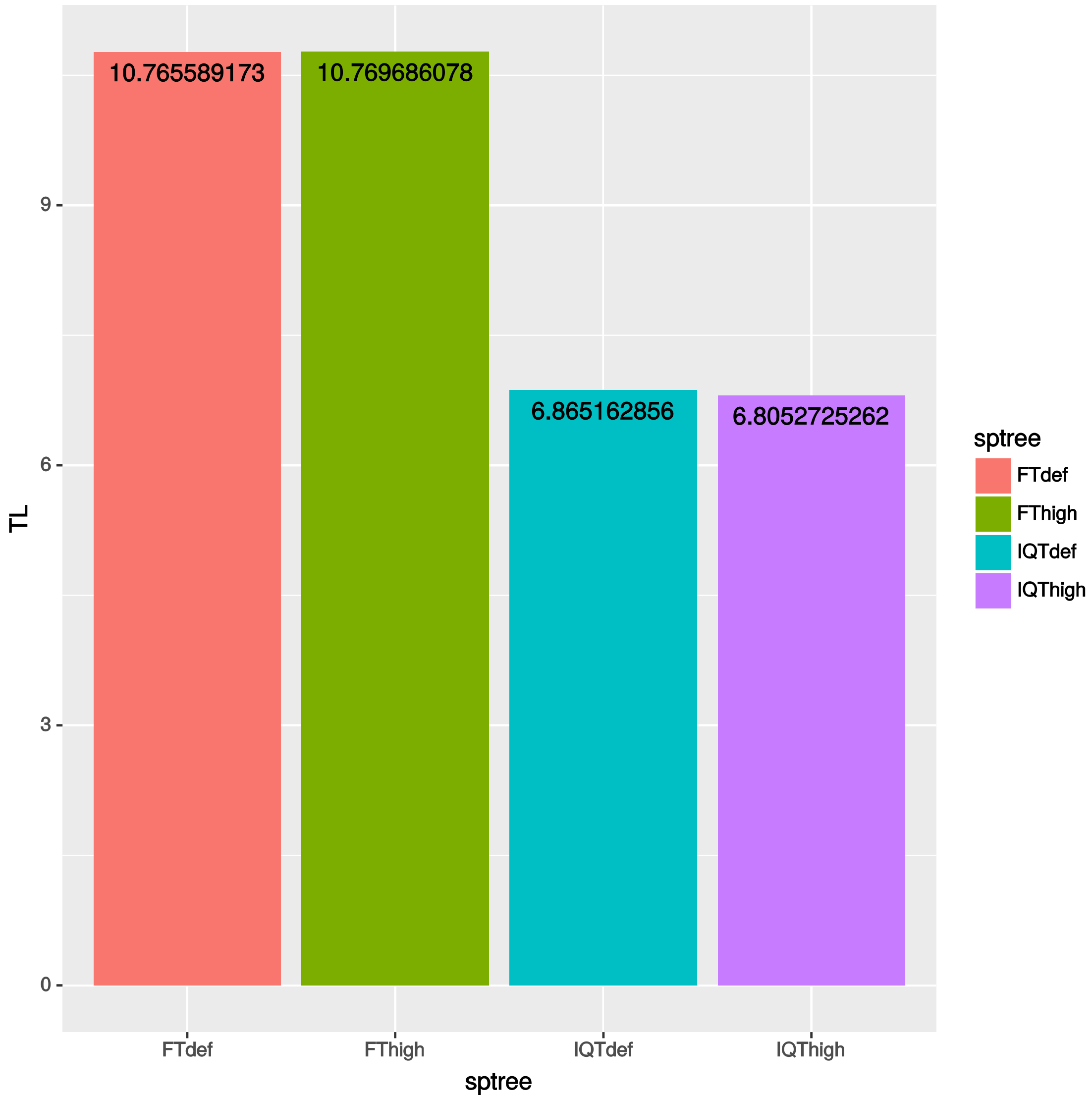

Figure S2. Barplot of the tree-lengths (summ of edge lengths) of each species-tree estimated by the indicated search types (see Table 2).

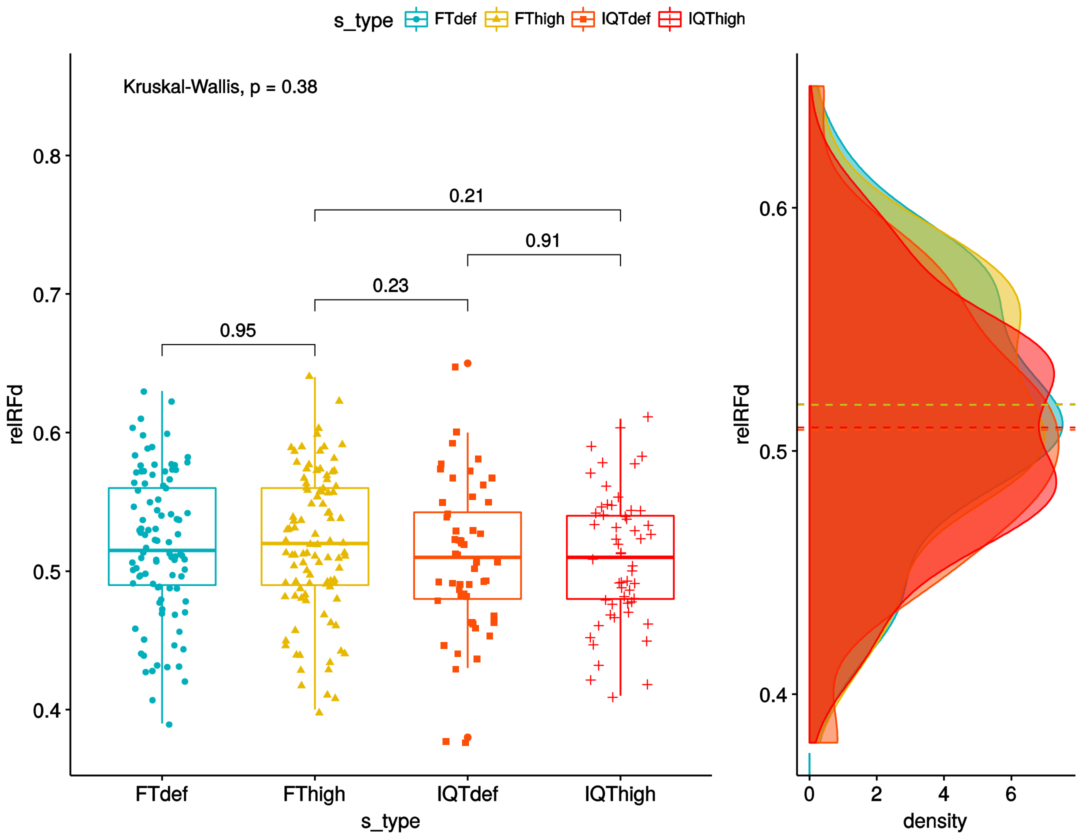

Figure S3. Distribution of Robinson-Foulds distances of gene trees to the corresponding species tree computed from the supermatrix of concatenated top-scoring markers by each search type.

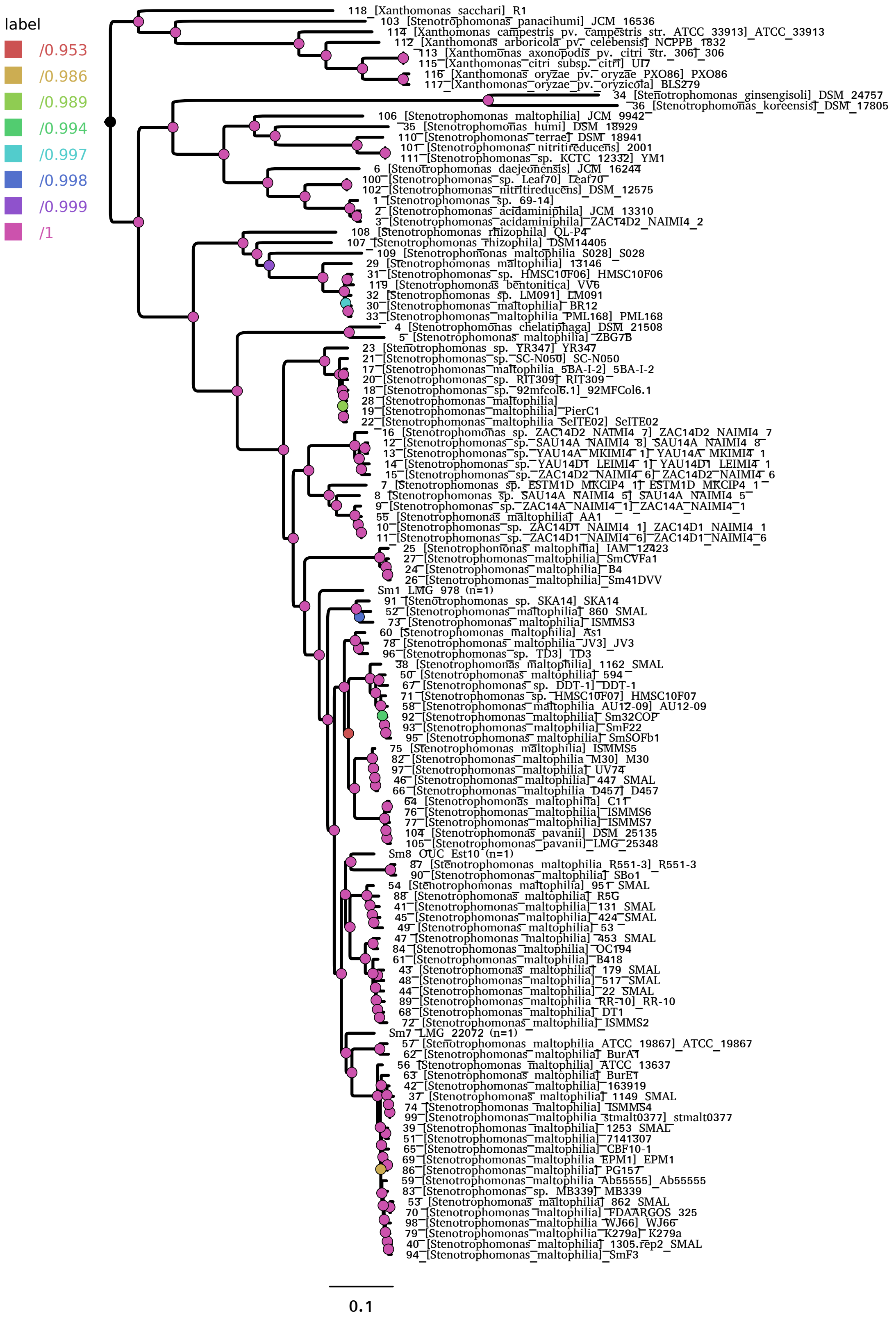

Figure S4. Full version of the IQ-TREE ML species tree for 119 genomes shown in Figure 5 of the main text.

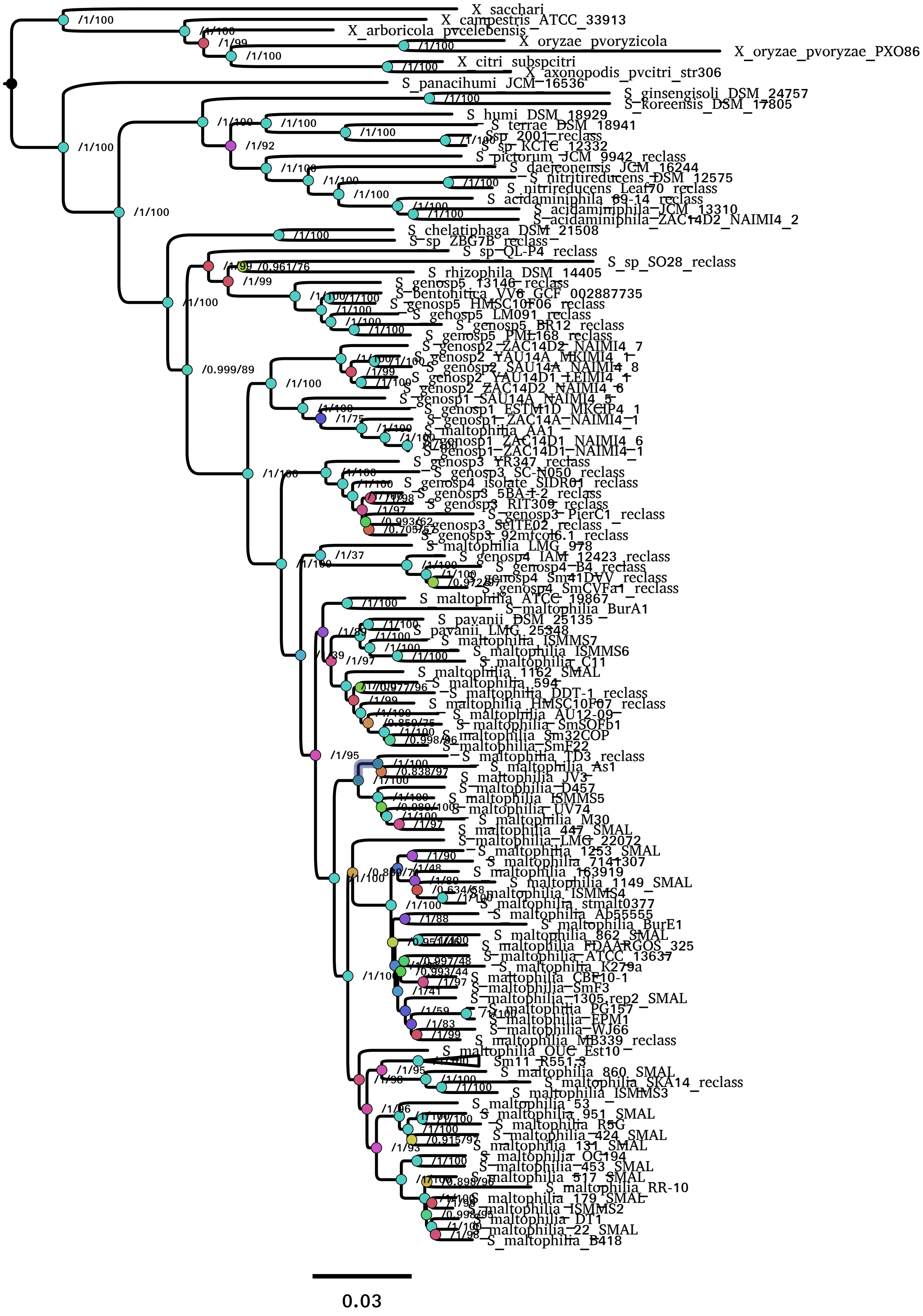

Figure S5. Full version of the ML pan-genome tree shown in Figure 6 of the main text

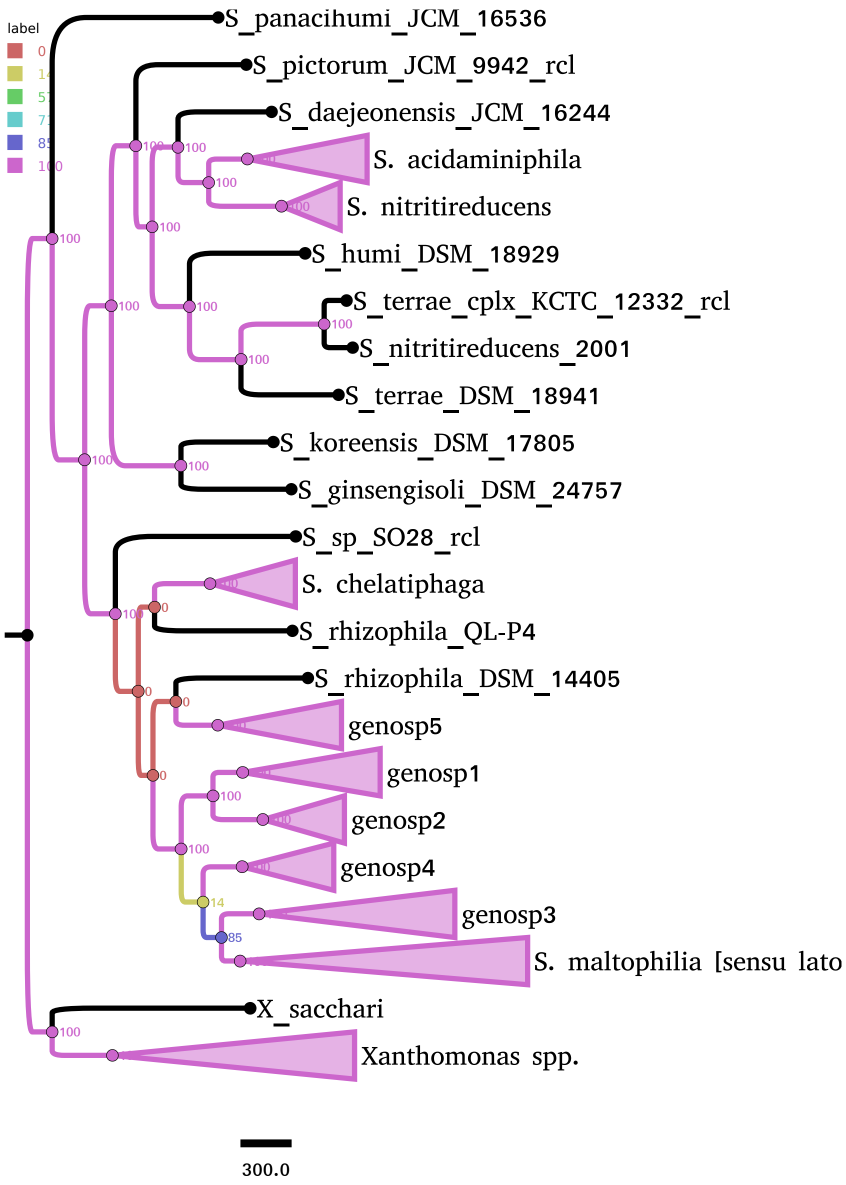

Figure S6. Best parsimony tree (score = 63977) found among 3200 independent pars searches + bootstrap analysis (4000 pseudoreplicates) performed on the PGM (31,433 consensus clusters computed from those identified by COGtriangles + OMC) for 118 selected *Stenotrophomonas* + *Xanthomonas* (outgroup) genomes.

2. Supplementary tables

Table S1. Overview of key characteristics of the genome assemblies reported in this study for 10 environmental isolates recovered from Mexican rivers and classified as genospecies 1 and 2 in the study of Ochoa-Sánchez and Vinuesa (2017). Details of their isolation sites and antimicrobial resistance phenotypes can be found therein. All genomes consist of a single gapped scaffold. The RefSeq acc. numbers and key annotation features for each genome are presented in Table 1 of the main text.

**Genome**	**Num_gaps**	**Num_Ns**	**Size_nt**	**BioProject**	**BioSample**
Stenotrophomonas genospecies1 ESTM1D MKCIP4 1	6	240	4475880	PRJNA429695	SAMN08357817
Stenotrophomonas genospecies1 SAU14A NAIMI4 5	9	180	4570883	PRJNA429695	SAMN08357818
Stenotrophomonas genospecies1 ZAC14A NAIMI4 1	11	575	4698328	PRJNA429695	SAMN08357819
Stenotrophomonas genospecies1 ZAC14D1 NAIMI4 1	3	1076	4702461	PRJNA429695	SAMN08357820
Stenotrophomonas genospecies1 ZAC14D1 NAIMI4 6	11	920	4700343	PRJNA429695	SAMN08357821
Stenotrophomonas genospecies2 SAU14A NAIMI4 8	4	369	4479100	PRJNA429695	SAMN08357822
Stenotrophomonas genospecies2 YAU14A MKIMI4 1	9	1361	4487007	PRJNA429695	SAMN08357823
Stenotrophomonas genospecies2 YAU14D1 LEIMI4 1	5	193	4319112	PRJNA429695	SAMN08357824
Stenotrophomonas genospecies2 ZAC14D2 NAIMI4 6	9	347	4431104	PRJNA429695	SAMN08357825
Stenotrophomonas genospecies2 ZAC14D2 NAIMI4 7	13	423	4468731	PRJNA429695	SAMN08357826

Table S2. Genomic characteristics of the selected reference genomes after excluding the highly fragmented ones (> 300 contigs).

Genus	Species	Reclass	Strain	Fragments	Size_nt	Status	BioProject	BioSample	Assembly	PMID
Stenotrophomonas	*acidaminiphila*	Y	69-14	27	3693706	draft	PRJNA279279	SAMN05660631		NA
Stenotrophomonas	*acidaminiphila*	N	JCM_13310	126	3942520	draft	PRJNA284377	SAMN03701729		27014232
Stenotrophomonas	*acidaminiphila*	N	ZAC14D2_NAIMI4_2	1	4138297	complete	PRJNA296415	SAMN04099006	GCA_001314305.1	26659678
Stenotrophomona	*bentonitica*	N	VV6	45	4364949	draft	PRJNA427609	SAMN08241739	GCA_002887735.1	29472346
Stenotrophomonas	*chelatiphaga*	Y	ZBG7B	145	4065399	draft	PRJNA272355	SAMN03280975		26659682
Stenotrophomonas	*chelatiphaga*	N	DSM_21508	148	3967734	draft	PRJNA284369	SAMN03701678		27014232
Stenotrophomonas	*daejeonensis*	N	JCM_16244	124	3284859	chromosome	PRJNA284378	SAMN03701730		27014232
Stenotrophomonas	genosp3	Y	5BA-I-2	4	4568054	draft	PRJNA224116	SAMN02641498	GCF_000543365.1	24604648
Stenotrophomonas	genosp3	Y	92mfcol6.1	11	4596228	draft	PRJNA224116	SAMN04488690	GCF_900116025.1	NA
Stenotrophomonas	genosp3	Y	PierC1	59	4638575	draft	PRJEB8824	SAMEA3309462		26276674
Stenotrophomonas	genosp3	Y	RIT309	45	4634795	draft	PRJNA224116	SAMN02676627	GCF_000632045.1	24812212
Stenotrophomonas	genosp3	Y	SC-N050	24	4484445	draft	PRJNA224116	SAMN05720615	GCF_900111475.1	NA
Stenotrophomonas	genosp3	Y	SeITE02	63	4557111	draft	PRJNA224116	SAMEA3138997	GCF_000613205.1	24812214
Stenotrophomonas	genosp3	Y	YR347	11	4686524	draft	PRJNA224116	SAMN05518671	GCF_900188015.1	NA
Stenotrophomonas	genosp4	Y	B4	180	4334733	draft	PRJNA224116	SAMN03753636	GCF_001276355.1	NA
Stenotrophomonas	genosp4	N	IAM_12423	9	4144460	draft	PRJNA224116	SAMN04487782	GCF_900113425.1	NA
Stenotrophomonas	genosp4	N	isolate_SIDR01	2	4602565	draft	PRJNA248909	SAMN06040735		NA
Stenotrophomonas	genosp4	Y	Sm41DVV	26	4139723	draft	PRJNA323790	SAMN05188789		NA
Stenotrophomonas	genosp4	Y	SmCVFa1	30	4264176	draft	PRJNA323845	SAMN05190067		NA
Stenotrophomonas	genosp5	Y	13146	60	4444179	draft	PRJNA224116	SAMN07237143	GCF_002205165.1	NA
Stenotrophomonas	genosp5	Y	BR12	80	4405362	draft	PRJNA224116	SAMN03456145	GCF_000972335.1	26472823
Stenotrophomonas	genosp5	Y	HMSC10F06	63	4114905	draft	PRJNA269850	SAMN03287020		NA
Stenotrophomonas	genosp5	Y	LM091	1	4317450	complete	PRJNA344031	SAMN05818440		NA
Stenotrophomonas	genosp5	Y	PML168	97	4403876	draft	PRJNA224116	SAMEA2272452	GCF_000308335.1	22887661
Stenotrophomonas	*ginsengisoli*	N	DSM_24757	99	3374112	draft	PRJNA284375	SAMN03701688		27014232
Stenotrophomonas	*humi*	N	DSM_18929	92	4122049	draft	PRJNA284364	SAMN03701675		27014232
Stenotrophomonas	*koreensis*	N	DSM_17805	58	3029904	draft	PRJNA284363	SAMN03701670		27014232
Stenotrophomonas	*maltophilia*	N	1149_SMAL	235	4847430	draft	PRJNA224116	SAMN03197105	GCF_001069295.1	26230489
Stenotrophomonas	*maltophilia*	N	1162_SMAL	108	4392826	draft	PRJNA224116	SAMN03197119	GCF_001068765.1	26230489
Stenotrophomonas	*maltophilia*	N	1253_SMAL	102	4833739	draft	PRJNA224116	SAMN03197220	GCF_001068915.1	26230489
Stenotrophomonas	*maltophilia*	N	1305.rep2_SMAL	158	5024838	draft	PRJNA224116	SAMN03197279	GCF_001069615.1	26230489
Stenotrophomonas	*maltophilia*	N	131_SMAL	71	4794818	draft	PRJNA224116	SAMN03197286	GCF_001069645.1	26230489
Stenotrophomonas	*maltophilia*	N	163919	148	4695069	draft	PRJNA224116	SAMN07237142	GCF_002205175.1	NA
Stenotrophomonas	*maltophilia*	N	179_SMAL	67	4533188	draft	PRJNA224116	SAMN03197369	GCF_001071655.1	26230489
Stenotrophomonas	*maltophilia*	N	22_SMAL	38	4457527	draft	PRJNA224116	SAMN03197412	GCF_001071815.1	26230489
Stenotrophomonas	*maltophilia*	N	424_SMAL	68	5202479	draft	PRJNA224116	SAMN03197617	GCF_001072915.1	26230489
Stenotrophomonas	*maltophilia*	N	447_SMAL	47	4802736	draft	PRJNA224116	SAMN03197640	GCF_001071115.1	26230489
Stenotrophomonas	*maltophilia*	N	453_SMAL	28	4779898	draft	PRJNA224116	SAMN03197645	GCF_001073045.1	26230489
Stenotrophomonas	*maltophilia*	N	517_SMAL	108	4569872	draft	PRJNA224116	SAMN03197711	GCF_001073225.1	26230489
Stenotrophomonas	*maltophilia*	N	53	127	4637887	draft	PRJNA260977	SAMN03067892		25883296
Stenotrophomonas	*maltophilia*	N	594	119	4484627	draft	PRJNA224116	SAMN07237146	GCF_002205215.1	NA
Stenotrophomonas	*maltophilia*	N	7141307	129	4725889	draft	PRJNA224116	SAMN07237141	GCF_002205295.1	NA
Stenotrophomonas	*maltophilia*	N	860_SMAL	266	4466361	draft	PRJNA224116	SAMN03198069	GCF_001074685.1	26230489
Stenotrophomonas	*maltophilia*	N	862_SMAL	238	4445747	draft	PRJNA224116	SAMN03198070	GCF_001074705.1	26230489
Stenotrophomonas	*maltophilia*	N	951_SMAL	259	4814445	draft	PRJNA224116	SAMN03198171	GCF_001075475.1	26230489
Stenotrophomonas	*maltophilia*	N	AA1	1	4663337	complete	PRJNA224116	SAMN06130959	GCF_002025605.1	28275097
Stenotrophomonas	*maltophilia*	N	Ab55555	6	4918930	draft	PRJNA164599	SAMN02596924		NA
Stenotrophomonas	*maltophilia*	N	As1	33	4394082	draft	PRJNA224116	SAMN03491122	GCF_001051925.1	26966198
Stenotrophomonas	*maltophilia*	N	ATCC_13637	182	4951541	draft	PRJNA224116	SAMN05721779	GCF_001997185.1	NA

Stenotrophomonas	*maltophilia*	N	ATCC_19867	14	4424025	draft	PRJNA224116	SAMN02441648	GCF_000382065.1	NA
Stenotrophomonas	*maltophilia*	N	AU12-09	125	4547300	draft	PRJNA174752	SAMN02469852		23640378
Stenotrophomonas	*maltophilia*	N	B418	231	4688249	draft	PRJNA224116	SAMN03161950	GCF_000788095.1	25700397
Stenotrophomonas	*maltophilia*	N	BurA1	64	4360660	draft	PRJEB8824	SAMEA3309460		26276674
Stenotrophomonas	*maltophilia*	N	BurE1	48	4504590	draft	PRJEB8824	SAMEA3309461		26276674
Stenotrophomonas	*maltophilia*	N	C11	242	4770820	draft	PRJNA285410	SAMN03753638		NA
Stenotrophomonas	*maltophilia*	N	CBF10-1	115	4556616	draft	PRJNA312840	SAMN04507546		NA
Stenotrophomonas	*maltophilia*	N	D457	1	4769156	complete	PRJEA89665	SAMEA2272378		22689246
Stenotrophomonas	*maltophilia*	Y	DDT-1	97	4514569	draft	PRJNA286061	SAMN03764479		26888254
Stenotrophomonas	*maltophilia*	N	DT1	64	4526952	draft	PRJNA224116	SAMN05915699	GCF_001866065.1	NA
Stenotrophomonas	*maltophilia*	N	EPM1	1	4787769	chromosome	PRJNA224116	SAMN02471395	GCF_000344215.1	23599297
Stenotrophomonas	*maltophilia*	N	FDAARGOS_325	1	4851139	complete	PRJNA224116	SAMN06173338	GCF_002208885.1	NA
Stenotrophomonas	*maltophilia*	Y	HMSC10F07	158	4491970	draft	PRJNA269851	SAMN03287021		NA
Stenotrophomonas	*maltophilia*	N	ISMMS2	1	4509724	complete	PRJNA277366	SAMN03389647		26324280
Stenotrophomonas	*maltophilia*	N	ISMMS3	1	4804002	complete	PRJNA277366	SAMN03389650		26324280
Stenotrophomonas	*maltophilia*	N	ISMMS4	3	4752108	draft	PRJNA224116	SAMN03389651	GCF_001275085.1	26324280
Stenotrophomonas	*maltophilia*	N	ISMMS5	18	4784825	draft	PRJNA224116	SAMN03389652	GCF_001275075.1	26324280
Stenotrophomonas	*maltophilia*	N	ISMMS6	10	4832466	draft	PRJNA224116	SAMN03389653	GCF_001275095.1	26324280
Stenotrophomonas	*maltophilia*	N	ISMMS7	2	4702773	draft	PRJNA224116	SAMN03389654	GCF_001651505.1	26324280
Stenotrophomonas	*maltophilia*	N	JV3	1	4544477	complete	PRJNA53943	SAMN02261377		NA
Stenotrophomonas	*maltophilia*	N	K279a	1	4851126	complete	PRJNA30351	SAMEA1705934		18419807
Stenotrophomonas	*maltophilia*	N	LMG_22072	173	4512166	draft	PRJNA224116	SAMN04207869	GCF_001431675.1	27014232
Stenotrophomonas	*maltophilia*	N	LMG_978	109	4484616	draft	PRJNA224116	SAMN04207868	GCF_001431665.1	27014232
Stenotrophomonas	*maltophilia*	N	M30	193	4902008	draft	PRJNA235918	SAMN02592618		24926059
Stenotrophomonas	*maltophilia*	Y	MB339	262	4897648	draft	PRJNA224116	SAMN06176942	GCF_001974685.1	NA
Stenotrophomonas	*maltophilia*	N	OC194	202	4688045	draft	PRJNA224116	SAMN04041569	GCF_001297005.1	NA
Stenotrophomonas	*maltophilia*	N	OUC_Est10	1	4668743	complete	PRJNA224116	SAMN04992827	GCF_002138415.1	NA
Stenotrophomonas	*maltophilia*	N	PG157	76	4949420	draft	PRJNA295129	SAMN04260440		NA
Stenotrophomonas	*maltophilia*	N	R551-3	1	4573969	complete	PRJNA224116	SAMN00623065	GCF_000020665.1	NA
Stenotrophomonas	*maltophilia*	N	R5G	79	4992669	draft	PRJNA329467	SAMN05413110		NA
Stenotrophomonas	*maltophilia*	N	RR-10	158	4660562	draft	PRJNA224116	SAMN02471024	GCF_000237025.1	22328769
Stenotrophomonas	*maltophilia*	N	SBo1	23	4800701	draft	PRJNA326914	SAMN05294119		NA
Stenotrophomonas	*maltophilia*	Y	SKA14	3	5020454	draft	PRJNA19369	SAMN02436221		NA
Stenotrophomonas	*maltophilia*	N	Sm32COP	45	4548960	draft	PRJNA323771	SAMN05188742		NA
Stenotrophomonas	*maltophilia*	N	SmF22	64	4583062	draft	PRJNA323842	SAMN05190064		NA
Stenotrophomonas	*maltophilia*	N	SmF3	77	4595297	draft	PRJNA323803	SAMN05189120		NA
Stenotrophomonas	*maltophilia*	N	SmSOFb1	93	4483386	draft	PRJNA323844	SAMN05190066		NA
Stenotrophomonas	*maltophilia*	N	stmalt0377	120	4620840	draft	PRJNA224116	SAMEA3138820	GCF_000499565.1	NA
Stenotrophomonas	*maltophilia*	Y	TD3	10	4917215	draft	PRJNA224116	SAMN05898096	GCF_001939825.1	NA
Stenotrophomonas	*maltophilia*	N	UV74	179	4889583	draft	PRJNA261822	SAMN03076212		26067959
Stenotrophomonas	*maltophilia*	N	WJ66	75	4657282	draft	PRJNA229185	SAMN03334939		25654114
Stenotrophomonas	*nitritireducens*	Y	Leaf70	11	4032657	draft	PRJNA224116	SAMN04151613	GCF_001422025.1	26633631
Stenotrophomonas	*nitritireducens*	N	2001	1	4541729	complete	PRJNA224116	SAMN05428703	GCF_001700965.1	NA
Stenotrophomonas	*nitritireducens*	N	DSM_12575	95	3983487	draft	PRJNA284361	SAMN03701629		27014232
Stenotrophomonas	*panacihumi*	N	JCM_16536	141	3923155	draft	PRJNA299448	SAMN04207867		27014232
Stenotrophomonas	*pavanii*	N	DSM_25135	129	4313798	draft	PRJNA284376	SAMN03701690		27014232
Stenotrophomonas	*pavanii*	N	LMG_25348	17	4424001	draft	PRJNA224116	SAMN04487784	GCF_900101175.1	NA
Stenotrophomonas	*pictorum*	N	JCM_9942	829	3508292	draft	PRJNA299446	SAMN04207798	GCF_001310775.1	27014232
Stenotrophomonas	*rhizophila*	N	DSM_14405	1	4648976	complete	PRJNA244760	SAMN02727981		NA
Stenotrophomonas	*rhizophila*	N	QL-P4	1	4198652	complete	PRJNA326321	SAMN05276013		NA
Stenotrophomonas	*sp.*	Y	SO28	297	3754755	draft	PRJNA224116	SAMN02469568	GCF_000295735.1	23144428
Stenotrophomonas	*terrae*	Y	KCTC_12332	1	4541594	complete	PRJNA310387	SAMN04451766		NA
Stenotrophomonas	*terrae*	N	DSM_18941	143	4410319	draft	PRJNA284366	SAMN03701677		27014232
Xanthomonas	*arboricola*	N	pvcelebensis	3	4908017	draft	PRJNA254393	SAMN02903333		NA

Xanthomonas	*axonopodis*	N	pvcitri_str306	3	5274174	complete	PRJNA297	SAMN02603846		12024217
Xanthomonas	*campestris*	N	ATCC_33913	1	5076188	complete				12024217
Xanthomonas	*citri*	N	subspcitri	3	5222287	complete	PRJNA255042	SAMN02911839		25689023
Xanthomonas	*oryzae*	N	pvoryzae_PXO86	1	5016623	complete	PRJNA237250	SAMN02616002		NA
Xanthomonas	*oryzae*	N	pvoryzicola	1	4790622	complete	PRJNA280380	SAMN03612248		NA
Xanthomonas	*sacchari*	N		2	5006106	complete	PRJNA271115	SAMN03273272		NA

3. Supplementary code snippets, as examples on how to run a selection of the auxiliary scripts distributed in the GET_PHYLOMARKERS package.

# 1. Launch parallel IQ-TREE runs on all alignments with “fasta” extension name present in the current directory to find best-fitting model from the specified model set and then estimate the ML phylogeny using the -fast mode implented in verstion 1.6.1. Estimate SH-alrt branch support values during the search.

```
run_parallel_cmmds.pl fasta 'iqtree -s $file -st DNA -mset
"HKY,TN,TVM,TIM,TIM2,TIM3,GTR" -m MFP -nt 1 -alrt 1000 -fast'
```

# 2. Parse the ln*L* scores of the gene-trees the lnL scores of the gene-trees found by IQ-TREE and write them to a file with tab-separated values format.

```
 grep '^BEST SCORE' *log | sort -nrk5 | cut -d: -f1,3 | perl -pe 's/\h+/\t/; s/://'
> sorted_IQT_lnL_mset_gene_trees.tsv
```

# 3. Launch parallel FastTree runs on all alignments with “fasta” extension name present in the current directory using default tree search intensity and parse the lnL scores of the resulting gene-trees.

```
run_parallel_cmmds.pl fasta 'FastTree -nt -gtr -gamma -slow -slownni -spr 16
-sprlength 10 -log ${file%.*}_FT.log < $file > ${file%.*}_FTGTRG.ph'

grep '^Gamma20LogLk' *defFT.log | cut -f1,2 | sed 's/:Gamma20LogLk//; s/ /\t/' >
FT_lnL_defFT.tsv
```

# 4 Compute the average support values and Robinson-Foulds distances of gene trees to a species-tree

```
compute_suppValStas_and_RF-dist.R '/full/path/to/genetrees/' 1 fasta ph 1
```

# 5. Run multiple iqtree searches on the concatenated supermatrix using the best substitution model on multiple nodes by launching 50 subshels. (This was run on a 64 core server with 0.5 Tb of RAM).

```
for rep in {1..50}
do
   nohup nice iqtree -s concat_cdnAlns.fnainf -st DNA -m GTR+F+ASC+R6 -abayes -nt 1
-pre abayes_run${rep} &> /dev/null &
done
```

# 6. Parse FastTree ln*L* scores and computation times from logfiles (multiple independent runs)

```
grep '^Gamma20LogLk' *FTGTRG_slow_slownni_sprl10_spr10.log | cut -f1,2 | sed \
 's/:Gamma20LogLk//; s/ /\t/' > FT_lnL_cdnAln_FTGTRG_slow_slownni_sprl10_spr10.tsv

grep '^Total time:' *FTGTRG_slow_slownni_sprl10_spr10.log | cut -d' ' -f3 \
 > FT_total_wall_clock_seconds_cdnAln_FTGTRG_slow_slownni_sprl10_spr10.tsv
```

# 7. Parse IQ-TREE ln*L* scores and computation times from logfiles

```
grep '^BEST SCORE' *log | sort -nrk5 | cut -d: -f1,3 | perl -pe 's/\h+/\t/; s/://'
> sorted_IQT_lnL_mset_gene_trees.tsv

grep '^BEST SCORE' *log | cut -d: -f1,3 | perl -pe 's/\h+/\t/; s/://' >
sorted_by_FILE_NAME_lnL_mset_gene_trees.tsv

grep 'Total wall-clock' *log | cut -d: -f1,3 | perl -pe 's/:\h+/\t/; s/\s+sec.*$//'
> total_wall_clock_seconds_IQT_mset_MFP.tsv
```

Supplementary References

Luz Edith Ochoa-Sanchez and Pablo Vinuesa (2017). Evolutionary genetic analysis uncovers multiple species with distinct habitat preferences and antibiotic resistance phenotypes in the *Stenotrophomonas maltophilia* complex. Front. Microbiol. 8: 1548. https://doi.org/10.3389/fmicb.2017.01548 | PubMed PMID: 28861062.
